# Exploration and validation of a novel signature of seven necroptosis-related genes to improve the clinical outcome of hepatocellular carcinoma

**DOI:** 10.1186/s12885-023-11521-x

**Published:** 2023-10-24

**Authors:** Qiqi Tao, Zhichao Lang, Yifei Li, Yuxiang Gao, Lifan Lin, Zhengping Yu, Jianjian Zheng, Suhui Yu

**Affiliations:** 1 Key Laboratory of Diagnosis and Treatment of Severe Hepato-Pancreatic Diseases of Zhejiang Province, The First Affiliated Hospital of Wenzhou Medical University, Wenzhou, China; 2 Department of Hepatobiliary Surgery, The First Affiliated Hospital of Wenzhou Medical University, No.2 fuxue lane, Wenzhou, Zhejiang P.R. China; 3 Key Laboratory of Clinical Laboratory Diagnosis and Translational Research of Zhejiang Province, The First Affiliated Hospital of Wenzhou Medical University, No.2 fuxue lane, Wenzhou, Zhejiang P.R. China

**Keywords:** Necroptosis, Prognostic signature, Immune infiltration, Macrophages, USP21.

## Abstract

Necroptosis has been reported to be involved in cancer progression and associated with cancer prognosis. However, the prognostic values of necroptosis-related genes (NRGs) in hepatocellular carcinoma (HCC) remain largely unknown. This study aimed to build a signature on the basis of NRGs to evaluate the prognosis of HCC patients. In this study, using bioinformatic analyses of transcriptome sequencing data of HCC (n = 370) from The Cancer Genome Atlas (TCGA) database, 63 differentially expressed NRGs between HCC and adjacent normal tissues were determined. 24 differentially expressed NRGs were found to be related with overall survival (OS). Seven optimum NRGs, determined using Lasso regression and multivariate Cox regression analysis, were used to construct a new prognostic risk signature for predicting the prognosis of HCC patients. Then survival status scatter plots and survival curves demonstrated that the prognosis of patients with high-Riskscore was worse. The prognostic value of this 7-NRG signature was validated by the International Cancer Genome Consortium (ICGC) cohort and a local cohort (Wenzhou, China). Notably, Riskscore was defined as an independent risk factor for HCC prognosis using multivariate cox regression analysis. Immune infiltration analysis suggested that higher macrophage infiltration was found in patients in the high-risk group. Finally, enhanced 7 NRGs were found in HCC tissues by immunohistochemistry. In conclusion, a novel 7-NRG prognostic risk signature is generated, which contributes to the prediction in the prognosis of HCC patients for the clinicians.

**Supplementary Information:**

The online version contains supplementary material available at 10.1186/s12885-023-11521-x.

## Introduction

Hepatocellular carcinoma (HCC) occurs as one of the most prevalent malignancies worldwide [1]. The symptoms of early HCC are insidious, and the available surveillance tools and biomarkers fail to satisfy the clinical requirements for HCC diagnosis and prognosis prediction [2]. Meanwhile, the lack of curative treatment and predictive strategies for advanced HCC results in an increase in its mortality rate by 2%~3% per year [3, 4]. Therefore, the identification of new biomarkers for HCC prognosis is urgent.

Necroptosis is an alternative programmed cell death pattern when the normal apoptotic pathway is inhibited. Necroptosis has mechanistic resemblance to apoptosis and morphological resemblance to necrosis, respectively [5]. Increasing evidence has shown that necroptosis, as a new promising therapeutic target, functions in the progression of a wide range of human cancers [6, 7]. In addition, necroptosis may induce a strong adaptive immune response that inhibits tumor development [8, 9]. Generally, the expressions of the necroptosis pathway-related regulators are dysregulated in cancer cells [10–12]. Unfortunately, the prognostic values of necroptosis-related genes (NRGs) in HCC remain largely unclear.

Herein, a novel prognostic risk signature of 7 NRGs was generated using weighted gene co-expression network analysis (WGCNA), Cox proportional risk regression analysis, and least absolute shrinkage and selection operator (Lasso) analysis in The Cancer Genome Atlas (TCGA) cohort. Subsequently, the prognostic value of this 7-NRG signature was validated in TCGA cohort and the International Cancer Genome Consortium (ICGC) cohort as well as a local cohort. Our results demonstrated that this signature had a good predictive power in HCC prognosis.

## Materials and methods

### Data source

Transcriptome sequencing data, mutation data, and basic clinical information of 370 patients with HCC were obtained from TCGA database ( https://portal.gdc.cancer.gov ). NRGs were gained from the Kyoto Encyclopedia of Genes and Genomes (KEGG) database ( https://www.genome.jp/kegg) [13–15]. The complete gene names are shown in Table S1. Transcriptome sequencing data of 231 donor patients with HCC from Japan and related basic clinical information were downloaded from the ICGC Data Portal database (https://dcc.icgc.org). We collected a total of 100 surgically resected HCC tissues from the First Hospital of Wenzhou Medical University (FAHWMU), and performed Quantitative Real-Time PCR (qRT-PCR) as the local cohort. Specific clinical parameters for TCGA cohort, ICGC cohort and the local cohort were shown in Table 1.

**Table 1:** Specific clinical parameters for the TCGA cohort, ICGC cohort and local cohort

Clinical parameters	Variable	TCGA cohort (n = 370)	ICGC cohort (n = 231)	local cohort (n = 100)
age	<=60	177(47.84%)	49(21.21%)	24(24%)
	> 60	193(52.16%)	182(78.79%)	76(76%)
gender	FEMALE	121(32.7%)	61(26.41%)	32(32%)
	MALE	249(67.3%)	170(73.59%)	68(68%)
stage	Stage I	171(46.22%)	36(15.58%)	13(13%)
	Stage II	85(22.97%)	105(45.45%)	25(25%)
	Stage III	85(22.97%)	71(30.74%)	41(41%)
	Stage IV	5(1.35%)	19(8.23%)	15(15%)
	unknown	24(6.49%)	0(0%)	6(6%)
grade	G1	55(14.86%)	unknown	17(17%)
	G2	177(47.84%)		29(29%)
	G3	121(32.7%)		36(36%)
	G4	12(3.24%)		14(14%)
	unknow	5(1.35%)		4(4%)
Recurrent	Primary	unknown	unknown	85(85%)
	Recurrent			15(15%)
Vascular invasion	Invasion	unknown	unknown	45(45%)
	No Invasion			55(55%)
HBV Infection	Infection	unknown	unknown	70(70%)
	No Infection			30(30%)

### Construction of 7-NRG signature

Firstly, differentially expressed necroptosis-related genes (DENRGs) between HCC and adjacent normal tissues were screened out (|log_2_FC| >1). DENRGs were divided into different modules via WGCNA. Prognostic NRGs were screened by univariate cox regression analysis. The Lasso algorithm was applied to prognostic NRGs to exclude overfitting genes. Finally, multivariate cox analysis was used to construct 7-NRG signature. The necroptosis Riskscore was calculated as Riskscore = ${\sum }_{i=1}^{N}\left(\text{E}\text{x}\text{p}\left(\text{i}\right)\bullet \text{c}\text{o}\text{e}\left(\text{i}\right)\right)$. Exp(i) is the transcriptional value of the genes, and coe(i) is the biased regression coefficient of the genes derived from the multivariate cox analysis.

### Independent prognostic factor analysis, nomogram, and calibration plots

Using the univariate and multivariate cox regression analyses, the independent prognostic factors for HCC were identified. R package “rms” was used to develop nomogram. “rms” and “survival” packages were applied to plot calibration curves of the nomogram.

### Enrichment analysis

Gene Set Enrichment Analysis (GSEA) [16] was performed to analyze all TCGA patients through “c5.go.v7.5.1.symbols.gmt” and “c2.cp.kegg.v7.5.1.symbols.gmt” gene sets via “limma”, “org.Hs.eg.db”, “DOSE”, “clusterProfiler” and “enrichplot” packages. R packages “clusterProfiler”, “org.Hs.eg.db”, “enrichplot” and “ggplot2” were used for Gene Ontology (GO) enrichment analysis [17].

### Immune infiltration analyses

Single-sample gene set enrichment analysis (ssGSEA) [18] was utilized to estimate the relative infiltration characteristics of 16 immune cells and 13 immune related functions in the TCGA cohort. The ssGSEA was performed via “GSVA” package. R packages “limma”, “reshape2” and “ggpubr” were used to analyze the difference of immune-infiltrating cells between the high- and low-risk subgroups.

### Tumor mutational burden (TMB) analysis

Upstream analysis of whole-genome sequencing and whole-exome sequencing data was conducted using “mattool” R package. Somatic mutation analysis was used to perform a systematic analysis of TMB for individual HCC patients in TCGA cohort. The “getsamplsummary” and “getgenesumprice” functions were used to retrieve patient information and genetic information, respectively. Package “maftools” was used to analyze TMB differences between the high- and low-Riskscore subgroups.

### Immunohistochemistry

Immunohistochemistry was performed as previously described [19]. Briefly, the tissues were immersed in 4% formalin for fixation, and then the formalin-fixed tissue was degreased and rehydrated. Next, the sections, blocked in 10% BSA, were in the incubation with primary antibody at 4 °C for at least 12 h. Then, the sections were incubated with a horseradish secondary antibody for 30 min.

### Cell culture

The cell line Huh7 and HL-7702  were purchased from ATCC. Huh7 was cultured in DMEM medium with 10% fetal bovine serum (FBS) and 1% antibiotics. HL-7702 was cultured in RPMI-1640 medium with 10% FBS and 1% antibiotics. Cells were maintained in a 37℃ incubator with 5% CO_2_ [20].

### Cell transfection

Huh7 and HL-7702 cells were cultured in a 6-well plate with 8 × 10^4^ cells per well. When the cell density was near to 80%, si-NC, si-USP21, si-NRF1 packaged byLipofectamine™ 2000 ( Invitrogen) were transfected into cells at 37℃ for 6 h. Then fresh medium was replaced and cells were collected for subsequent experiments after 48 h of transfection [20].

### qRT-PCR analysis

Total RNA was isolated from Huh7 cells and HCC tissues as well as adjacent normal tissues using the Tiangen RNA extraction reagent kit. Each sample was reversely transcribed into complementary DNA (cDNA) using a reverse-transcription (RT) reagent kit (Takara Biotechnology Co., Ltd., Dalian, China). Then, Real-time PCR was performed using SYBR Premix ExTaq (Takara). GAPDH was used as endogenous controls for mRNAs [20]. HSP90AA1 forward, 5’- AGGAGGTTGAGACGTTCGC − 3’; HSP90AA1 reverse, 5’- AGAGTTCGATCTTGTTTGTTCGG − 3’. PPIA forward, 5’- CCCACCGTGTTCTTCGACATT − 3’; PPIA reverse, 5’- GGACCCGTATGCTTTAGGATGA − 3’. SQSTM1 forward, 5’- GCACCCCAATGTGATCTGC − 3’; SQSTM1 reverse, 5’- CGCTACACAAGTCGTAGTCTGG − 3’. HSP90AB1 forward, 5’- AGAAATTGCCCAACTCATGTCC − 3’; HSP90AB1 reverse, 5’- ATCAACTCCCGAAGGAAAATCTC − 3’. FAF1 forward, 5’- GAGATGATCCTGGCGGATTTTC − 3’; FAF1 reverse, 5’- AGGTCCTGGTATGGTCTCACC − 3’. PGAM5 forward, 5’- TCGTCCATTCGTCTATGACGC − 3’; PGAM5 reverse, 5’- GGCTTCCAATGAGACACGG − 3’. USP21 forward, 5’-GAATCCTCGTGCTCCATCTGA − 3’; USP21 reverse, 5’-CAGCTGGTATACAGGACTTCCG-3’. GAPDH forward, 5’- AAAGCCTGCCGGTGACTAAC − 3’; GAPDH reverse, 5’- GCCCAATACGACCAAATCAGA − 3’. The full information of primer used for qRT-PCR were listed in Table S2.

### Western blot analysis

The proteins from Huh7 cells were extracted using RIPA extraction buffer. The protein samples of interested were separated by 10% SDS-PAGE electrophoresis, and then transferred to PVDF membranes. The primary anti-USP21 (Invitrogen, PA5-11055) and anti-GAPDH (CST, #2118) were added in PVDF membranes and incubated overnight at 4 °C. Then, the second antibody was added and incubated at room temperature for 1 h [19].

### Cell proliferation assays

Cell Counting Kit-8 (CCK8) (Dojindo, Japan) was used for the assessment of cell proliferation. Cells were seeded into 96-well plate at a density of 2 × 10^3^/100 µl per well to incubate for 48 h. Then, 10 µl CCK8 solution were added to each well and maintained in a 37 °C incubator for 1 h. Finally, the absorbance of each well was measured at 450 nm [20].

### Cell migration assays

Migration assays were performed in a Transwell chemotaxis 24-well chamber with 8.0 μm pore polycarbonate membrane insert (CORNING, 3422). Briefly, 3 × 10^4^ cells were plated in the upper chamber with a non-coated membrane. After 24 h of incubation at 37 °C, migrating cells were fixed and stained with 20% methanol and 0.1% crystal violet dye. Migrated cells were counted and imaged with an inverted microscope.

### Statistical analysis

At the present study, R software (version 4.1.0) was utilized for statistical analysis. Data were presented as mean ± SD of at least three independent experiments, and differences between two groups were compared using student’s t-test. Rank correlations were assessed by the performance of spearman’s correlation coefficient test among different variables. The R package “survival” was used for survival analysis, and Kaplan-Meier (K-M) survival curves were used to display survival differences between different groups. Statistical p-values were subjected to two tailed tests, and p < 0.05 was considered as significance.

## Results

### Screening prognostic NRGs and constructing 7-NRG signature

**Fig. 1:**
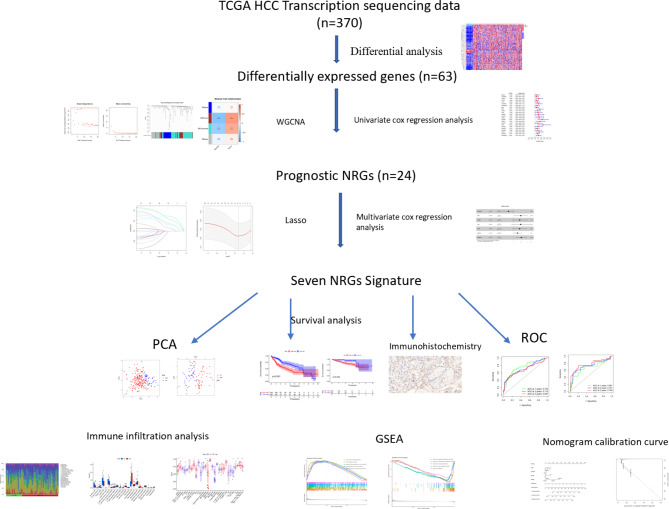
The general workflow of this study

**Fig. 2:**
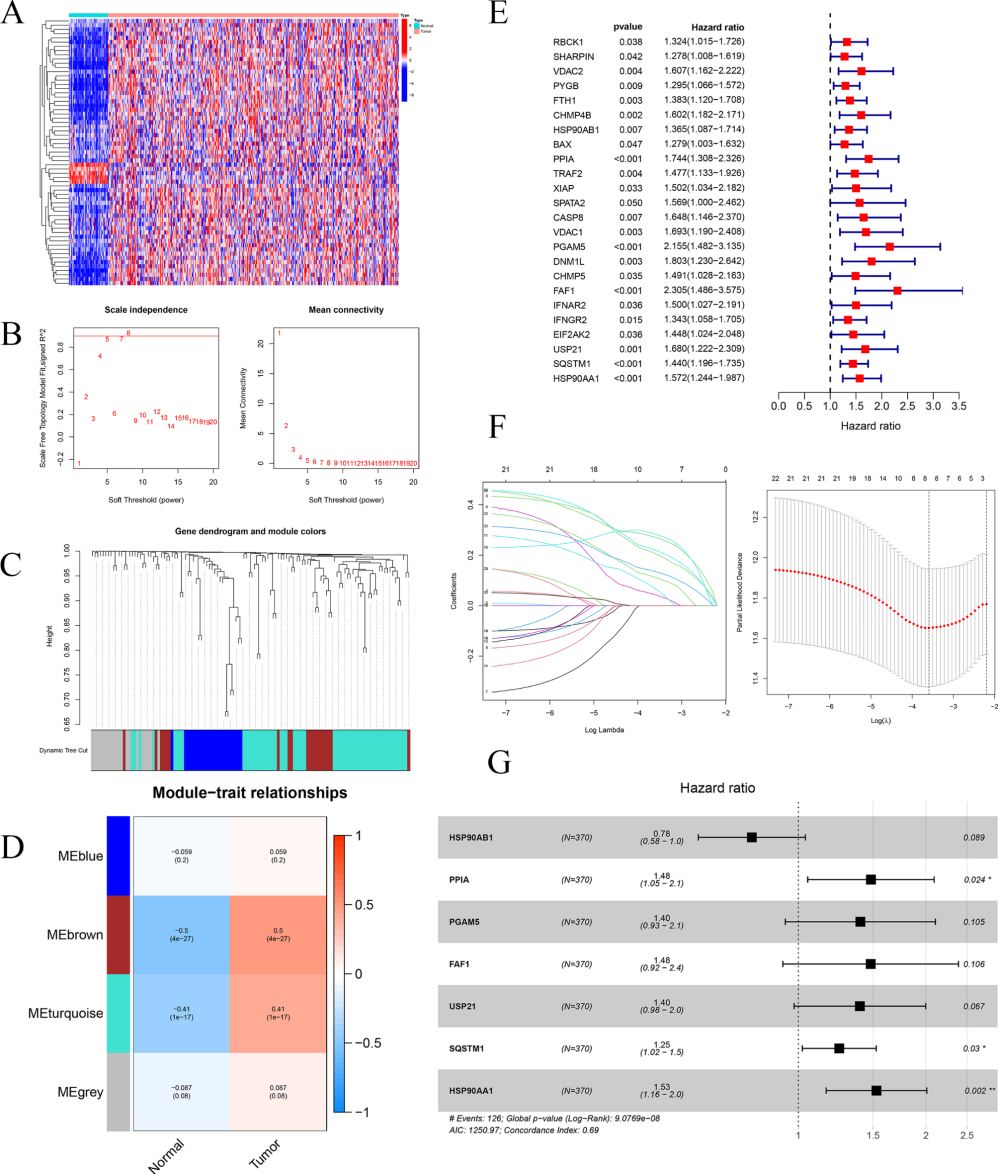
**Screening prognostic NRGs and seven NRGs signature construction**: (**A**) Heatmap of DENRGs between HCC tissues samples and adjacent normal samples. (**B**) Topology soft-thresholding analysis of network. (**C**) Clustering dendrogram of DENRGs. (**D**) Module-trait relationships. (**E**) Univariate cox regression analysis identified 24 prognostic DENRGs. (**F**) LASSO analysis for 24DENRGs. (**G**) Forest plot for multivariate cox regression analysis

The general workflow of this study was shown in Fig. 1. Firstly, the expressions of 130 NRGs were extracted from TCGA transcriptome sequencing data. Then DENRGs were identified between HCC tissues and adjacent normal tissues. Among DENRGs, 5 down-regulated and 58 up-regulated NRGs were found in HCC tissues (Fig. 2A**)**. 63 DENRGs were divided into different modules via WGCNA (Fig. 2B and D). MEbrown and MEturquoise modules-related genes, which were the most significant differentially expressed between HCC and adjacent normal tissues, were included in the next analysis. Subsequently, 24 DENRGs were identified as prognosis-related genes (Fig. 2E). 16 overfitted genes were excluded by Lasso algorithm (Fig. 2F). Multivariate cox regression analysis was utilized to construct 7-NRG signature (Fig. 2G). The Riskscore was calculated by the formula:

Riskscore = (HSP90AB1exp * -0.252) + (PPIA exp * 0.394) + (PGAM5 exp * 0.336) + (FAF1 exp * 0.393) + (USP21 exp * 0.334) + (SQSTM1 exp * 0.222) + (HSP90AA1 exp * 0.424).

### Validation of 7-NRG signature

**Fig. 3:**
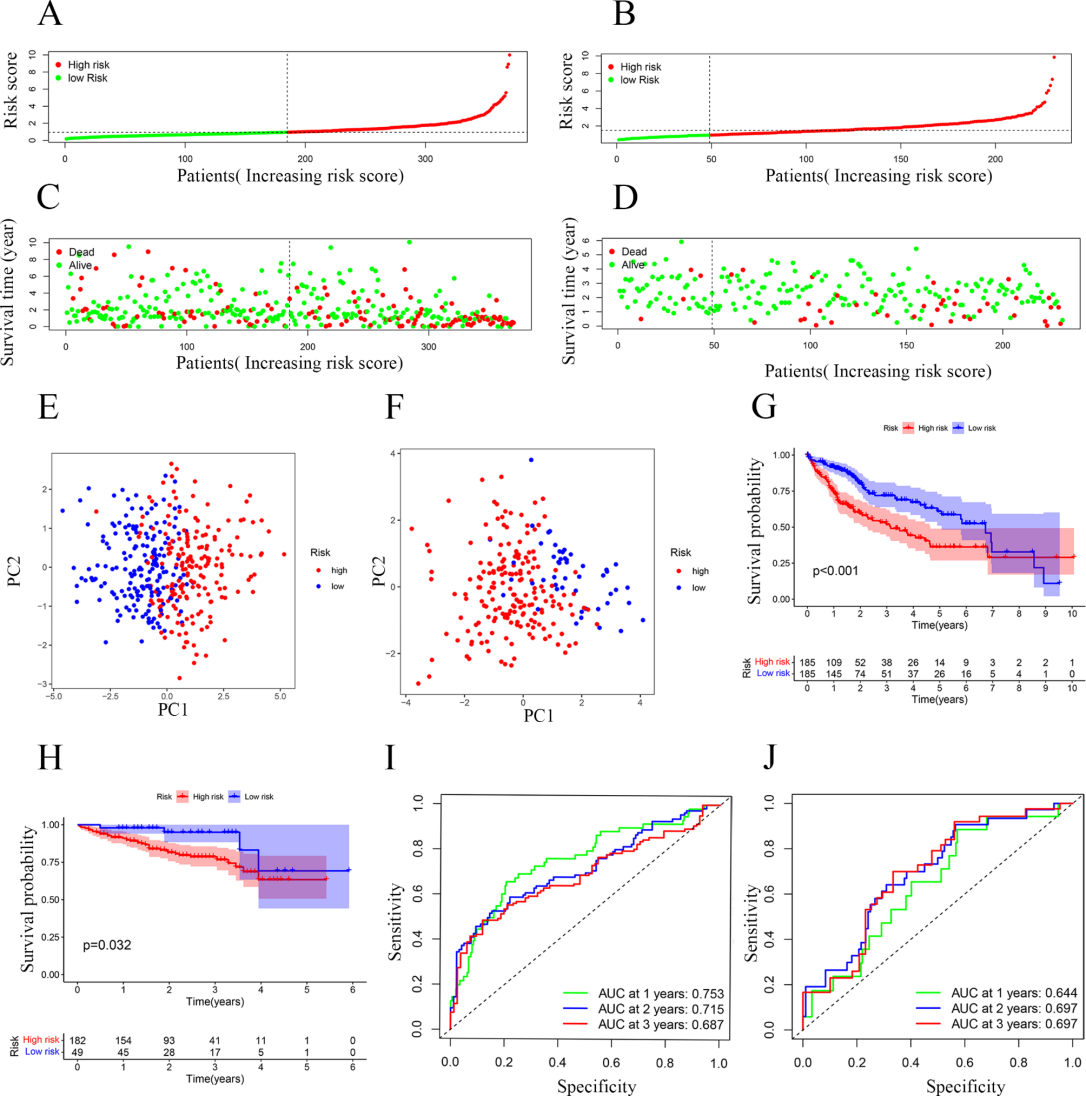
**Accuracy validation of seven NRGs signature** (**A**) Riskscores in TCGA cohort. (**B**) Riskscores in ICGC cohort. (**C**) Survival status scatterplot of patients in TCGA cohort. (**D**) Survival status scatterplot of patients in ICGC cohort. (**E**) Analysis of PCA for TCGA cohort. (**F**) Analysis of PCA for ICGC cohort. (**G**) Survival curves of patients in TCGA cohort. (**H**) Survival curves of patients in ICGC cohort. (**I**) ROC curve analysis for the value of prognosis in the signature for TCGA cohort. (**J**) ROC curve analysis for the value of prognosis in the signature for ICGC cohort

Patients in the TCGA cohort, the ICGC cohort, and the local cohort were divided into the high- and low-risk subgroups according to optimal Riskscore, respectively (Fig. 3A and B and Fig.S1A). Survival status scatterplot and K-M survival analyses suggested that patients with high-Riskscore had poorer survival than those with low-Riskscore (Fig. 3C and D and 3G, 3H, and Fig.S1B, S1D). The results of the principal component analysis (PCA) showed that the above dichotomous classification had a good performance (Fig. 3E and F and Fig.S1C). In the TCGA cohort, enhanced 7 NRGs were found in HCC tissues (Fig.S1F and Fig.S1G). Taken together, these results suggest that necroptosis functions in the development of HCC. The time-independent receiver operating characteristic curve (ROC) curve further demonstrated the prognostic value of this 7-NRG signature. As shown in Fig. 3I, the AUC value of the TCGA cohort reached 0.753 in the 1st year, 0.715 in the 2nd year, and 0.687 in the 3rd year, respectively. The AUC values of the ICGC cohort and the local cohort also demonstrated the accuracy of the 7-NRG signature in predicting HCC prognosis (Fig. 3J and Fig.S1E).

### Independent prognostic analysis, nomogram construction and calibration curves

**Fig. 4:**
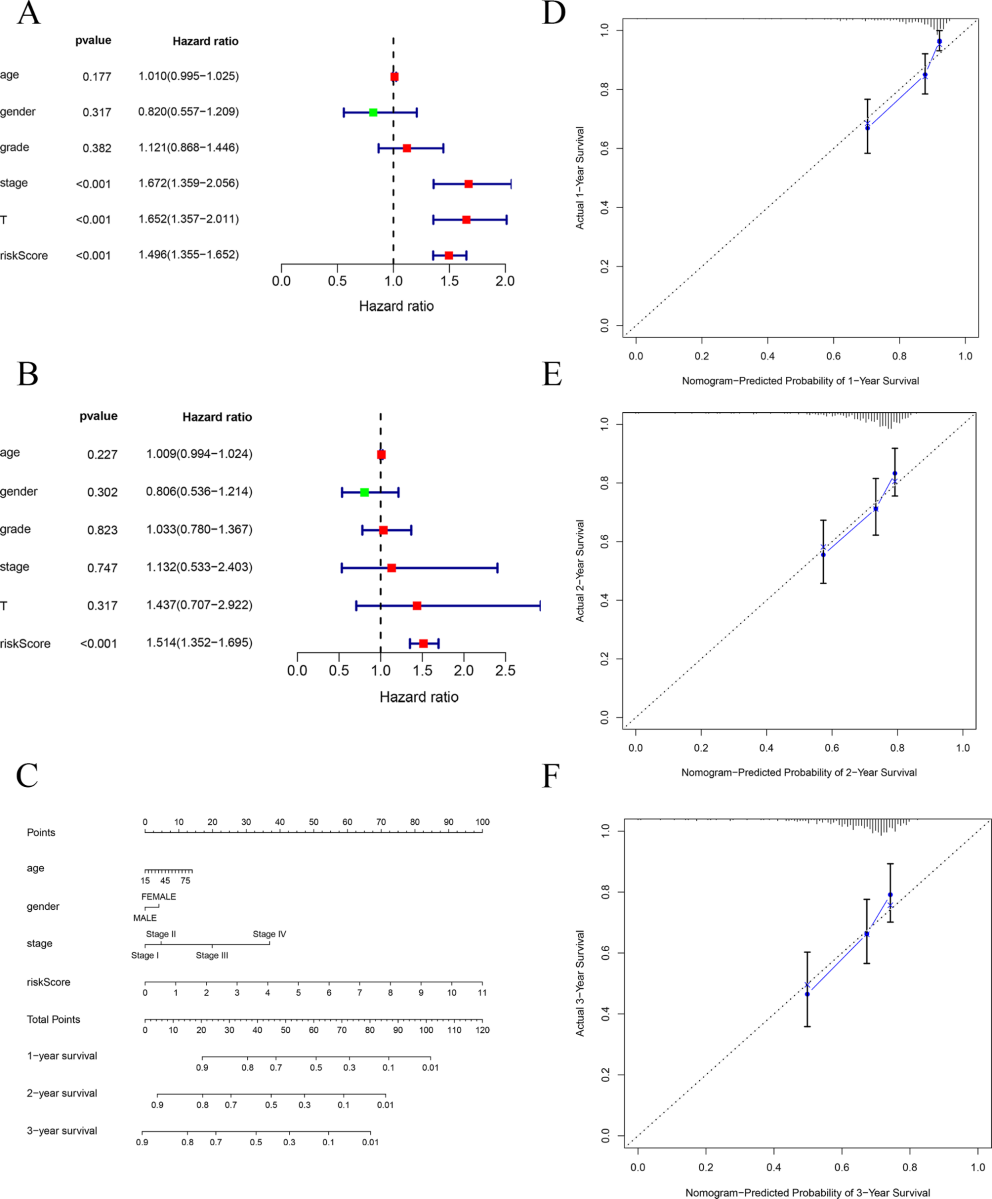
**Independent prognostic analysis, nomogram construction and calibration curves** (**A**) Univariate cox regression analyses. (**B**) Multivariate cox regression analyses. (**C**) Nomogram based on prognostic factors. (**D**-**F**) Calibration curve of the nomogram for the 1st, 2nd and 3rd year

To determine whether the Riskscore could serve as an independent risk factor for HCC, both univariate Cox analysis and multi-variate Cox analysis were performed. Univariate Cox analysis indicated that stage, T and Riskscore were prognostic factors for HCC (Fig. 4A). Further studies confirmed that only Riskscore was an independent prognostic factor for HCC via multivariate cox analysis (Fig. 4B). A novel clinical nomogram was constructed to precisely predict prognosis for HCC (Fig. 4C). The calibration curves demonstrated the excellent predictive performance of this nomogram (Fig. 4D-4F).

### Advanced HCC was associated with high Riskscore

**Fig. 5:**
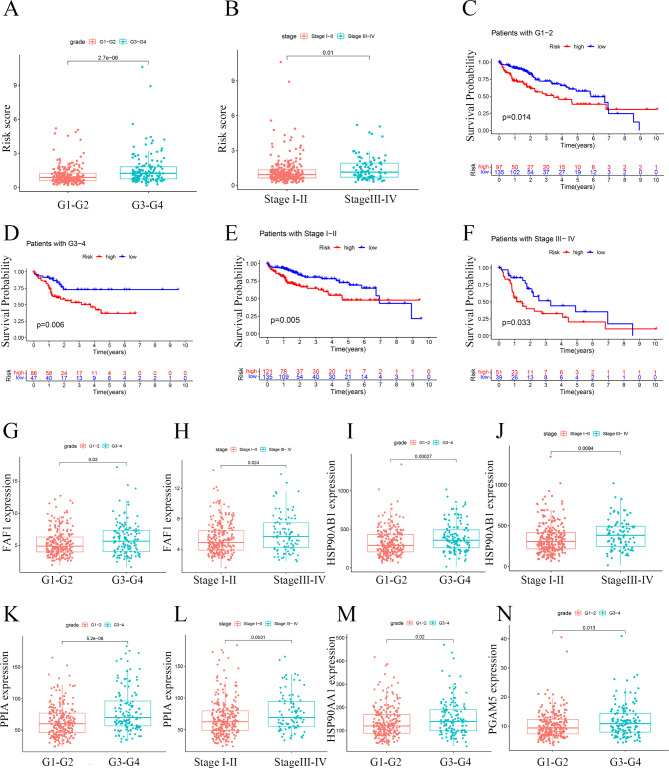
**Correlation analyses between Riskscore and clinical features** (**A**) Association between Riskscore and grade. (**B**) Association between Riskscore and stage. (**C**) Kaplan-Meier survival curves of G1-2 patients. (**D**) Kaplan-Meier survival curves of G3-4 patients. (**E**) Kaplan-Meier survival curves of patients with stage I-II. (**F**) Kaplan-Meier survival curves of patients with III-IV. (**G**) Correlation between grade and FAF1 expression. (**H**) Correlation between stage and FAF1 expression. (**I**) Correlation between grade and HSP90AB1expression. (**J**) Correlation between stage and HSP90AB1expression. (**K**) Correlation between grade and PPIA expression. (**L**) Association between stage and PPIA expression. (**M**) Correlation between grade and HSP90AA1 expression. (**N**) Correlation between grade and PGAM5 expression

Correlation analysis revealed that patients with high grade or stage was associated with high Riskscore (Fig. 5A and 5B). However, Riskscore was not correlated with age and gender (Fig.S2A and Fig.S2B). The survival curves suggested that high Riskscore was correlated with poor prognosis (Fig. 5C-5F). The correlation between Riskscore and the 7 NRGs expressions was subsequently explored. Except SQSTM1, the expressions of other 6 NRGs were correlated with grade (Fig. 5G, 5I, 5K, 5 M, 5 N and Fig.S2C). Stage was correlated with the expression of 3 NRGs including FAF1, HSP90AB1 and PPIA (Fig. 5H, 5J, 5 L). To conclude, advanced HCC may be associated with higher Riskscore. K-M survival analysis revealed that patients with upregulated FAF1, HSP90AA1, PPIA, PGAM5, USP21 and SQSTM1 genes may have shorter survival (Fig.S2D-S2I).

### Riskscore was correlated with immune cell infiltration

**Fig. 6:**
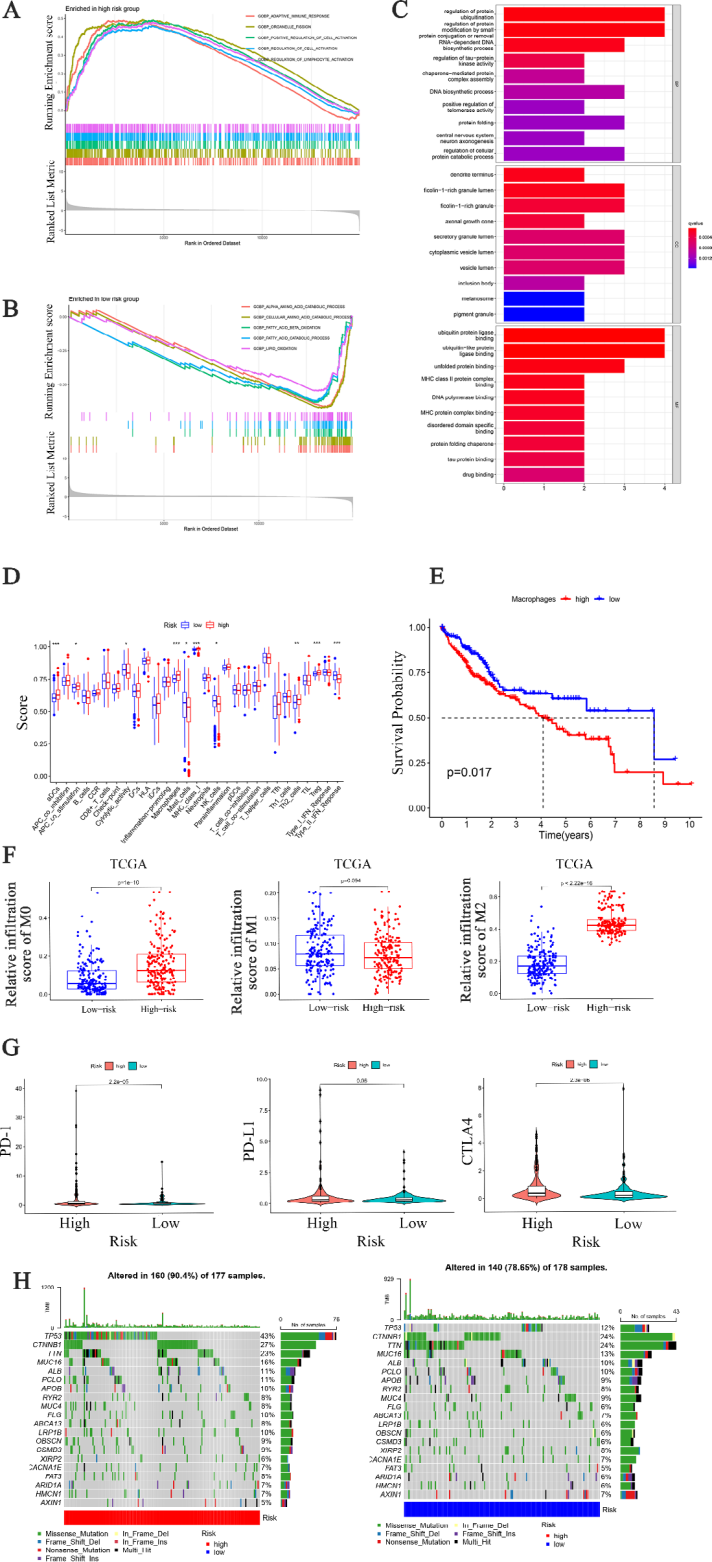
**Immune infiltration analysis** (**A**) GSEA enrichment analyses of high-Riskscore group. (**B**) GSEA enrichment analyses of low-Riskscore group. (**C**) The barplot of signature gene GO enrichment analysis. (**D**) Differential analyses of immune-related functions. Adjusted P values were demonstrated as: ns, namely, not significant; *P < 0.05; **P < 0.01; ***P < 0.001. (**E**) Survival curves of patients with different macrophages scores. (**F**) Relative infiltration of M0, M1 and M2 macrophages between high- and low-risk subgroups in TCGA cohort. (**G**) Relative levels of PD-1, PD-L1 and CTLA4 between high- and low-risk subgroups in TCGA cohort. (**H**) Tumor mutation burden between high- and low-risk subgroups in TCGA cohort

The correlation between Riskscore and immune cell infiltration was also analyzed. GSEA revealed that upregulated genes in the high-risk subgroup were mainly enriched in adaptive immunity response and cell activation biological processes (Fig. 6A). Moreover, upregulated genes in the low-risk subgroup were enriched in α-amino acid catabolism, fatty acid β-oxidation, fatty acid catabolism and lipid oxidation (Fig. 6B).

GO enrichment analysis uncovered that 7 NRGs were mainly involved in protein ubiquitination and deubiquitination (Fig. 6C). The relative infiltration characteristics of 16 immune cells and 13 immune related functions were analyzed in TCGA cohort. Higher macrophage infiltration was found in the high-risk subgroup than that in the low-risk subgroup (Fig. 6D). In addition, the scores of aDCs, APC_co_stimilation, Cytolytic_activity, Mast_cells, MHC_class_I, NK_cells, Th2_cells and Type_II_IFN_Reponse were obviously different between the high- and low-risk subgroups. Furthermore, K-M survival analysis revealed that the patients with high macrophage infiltration were correlated with worse prognosis (Fig. 6E). Furthermore, the infiltration scores of M0, M1 and M2 were evaluated between the high- and low-risk subgroups in the TCGA cohort. It was observed that patients in the high-risk subgroup had a higher M2 infiltration score (Fig. 6F). Compared with the low-risk subgroup, the high-risk subgroup had higher levels of PD1 and CTLA4 (Fig. 6G). The results of TMB analysis revealed that patients in the high-risk subgroup exhibit a higher frequency of TP53 mutation (Fig. 6H). These data suggest that high-risk patients may be more suitable candidates for immunotherapy.

#### 7 NRGs were upregulated in HCC tissues

**Fig. 7:**
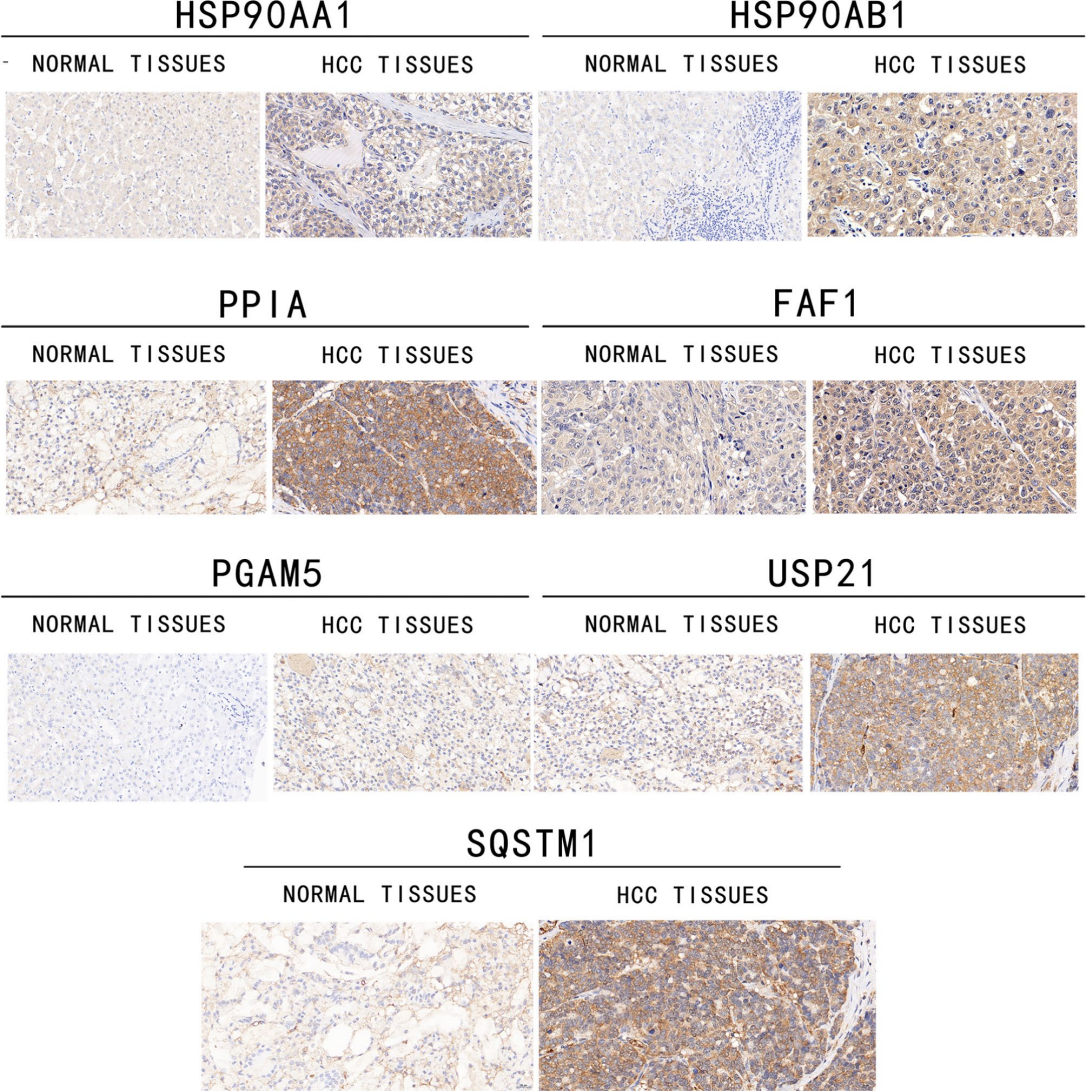
**Immunohistochemistry of 7 NRGs in HCC patients** 7 NRGs were enhanced in HCC tissues (n = 20) compared with adjacent tumor tissue samples (n = 20)

Immunohistochemistry analysis was performed to determine the protein expressions of 7 NRGs in 20 pairs of HCC and adjacent tumor tissue samples from the FAHWMU. Our data showed the upregulation of 7 NRGs in HCC tissues compared with adjacent normal tissues (Fig. 7), suggesting the possible oncogene roles of 7 NRGs in HCC. In addition, immunohistochemistry staining of PD-1 and PD-L1 was performed in the tumor samples from the FAHWMU. Clearly, patients with high-risk had enhanced levels of PD-1 and PD-L1 (Fig.S3).

### Transcriptional regulatory factor (TF) network construction

**Fig. 8:**
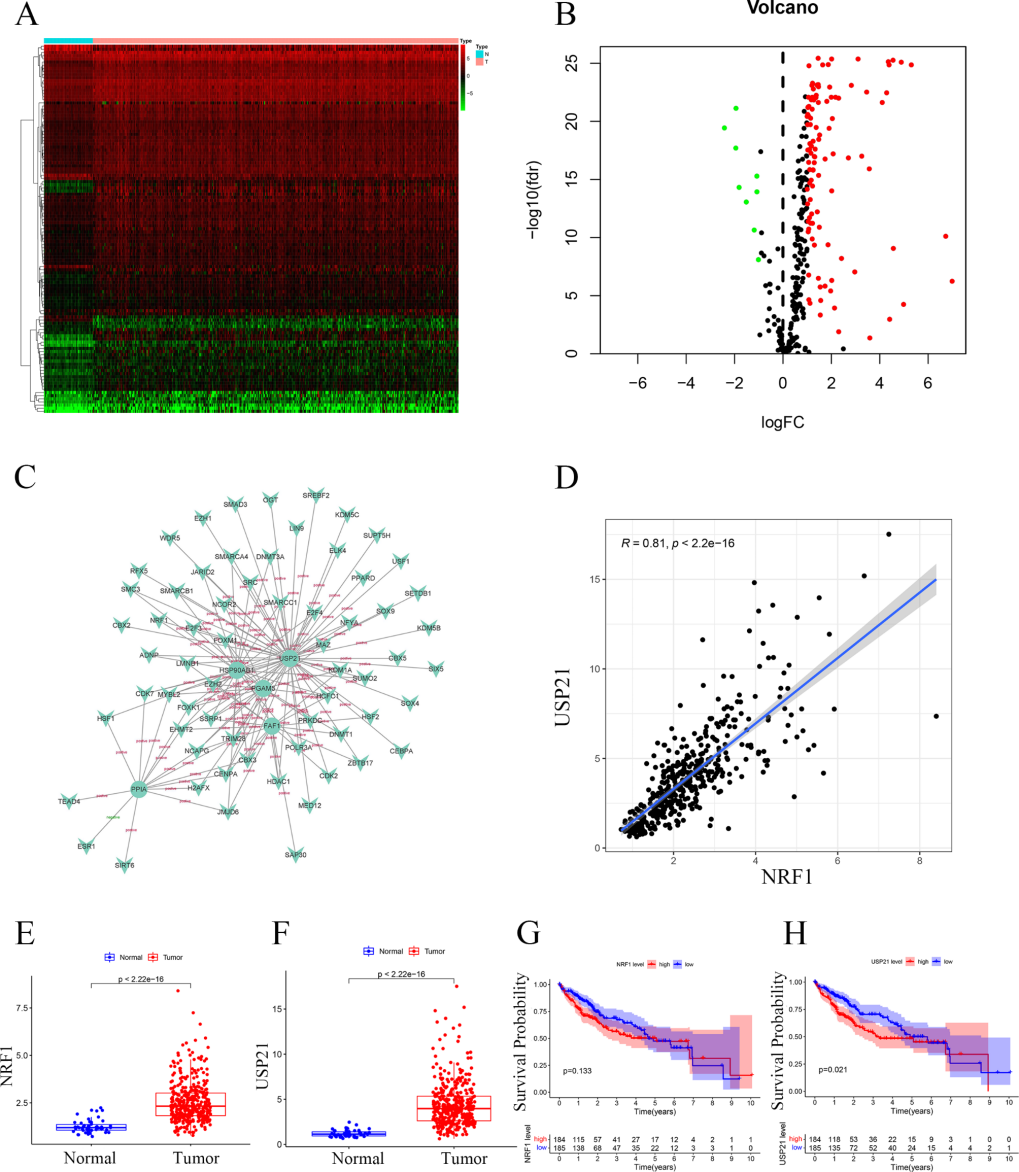
**TF network construction** (**A**) Heatmap of differentially expressed TFs. (**B**) Volcano plot of differentially expressed TFs. (**C**) TF network. Circular nodes represent NRGs, triangular nodes represent TFs. The line between nodes indicates a regulatory relationship between two nodes. (**D**) Co-expression relationship between NRF1 and USP21. (**E**) NRF1 expression. (**F**) USP21 expression. (**G**) Survival curves of patients with high- and low-NRF1. (**H**) Survival curves of patients with high- and low-USP21

Next, we explored the potential upstream regulation mechanisms of 7 NRGs via constructing TF network. Differential analysis of 318 cancer-related TFs was performed between HCC and adjacent normal tissues in the TCGA cohort (Fig. 8A and 8B). 117 eligible TFs (|log_2_FC| > 1, p < 0.05) were included in the construction of the TF network (Fig. 8C). Among the 7 NRGs, USP21 had the most TFs in the TF network. NRF1, which was the most correlated with USP21, was selected for the further analysis. It was found that the correlation coefficient between NRF1 and USP21 expression was 0.81 (Fig. 8D). In the TCGA cohort, both NRF1 and USP21 expressions were significantly upregulated in HCC tissues (Fig. 8E and F). Single-gene survival analysis showed no correlation between NRF1 expression and the OS of HCC patients (Fig. 8G). The prognosis of patients with high USP21 expression was worse than those with low USP21 (Fig. 8H). In the ICGC cohort, the correlation coefficient between NRF1 level and USP21 expression was 0.66 (Fig.S4A). In both the ICGC cohort and local cohort, the expression of NRF1 in HCC tissues was higher than that in adjacent normal tissues (Fig.S4B and Fig.S4D). Although there was no significant difference in survival rates between the high- and low-NRF1 group in both ICGC cohort and local cohort, data from the GEPIA2 database indicated that patients with high-NRF1 had shorter disease-free survival (Fig.S4C, S4E and S4F).

### NRF1 may enhances HCC proliferation and migration through upregulating USP21 expression

**Fig. 9:**
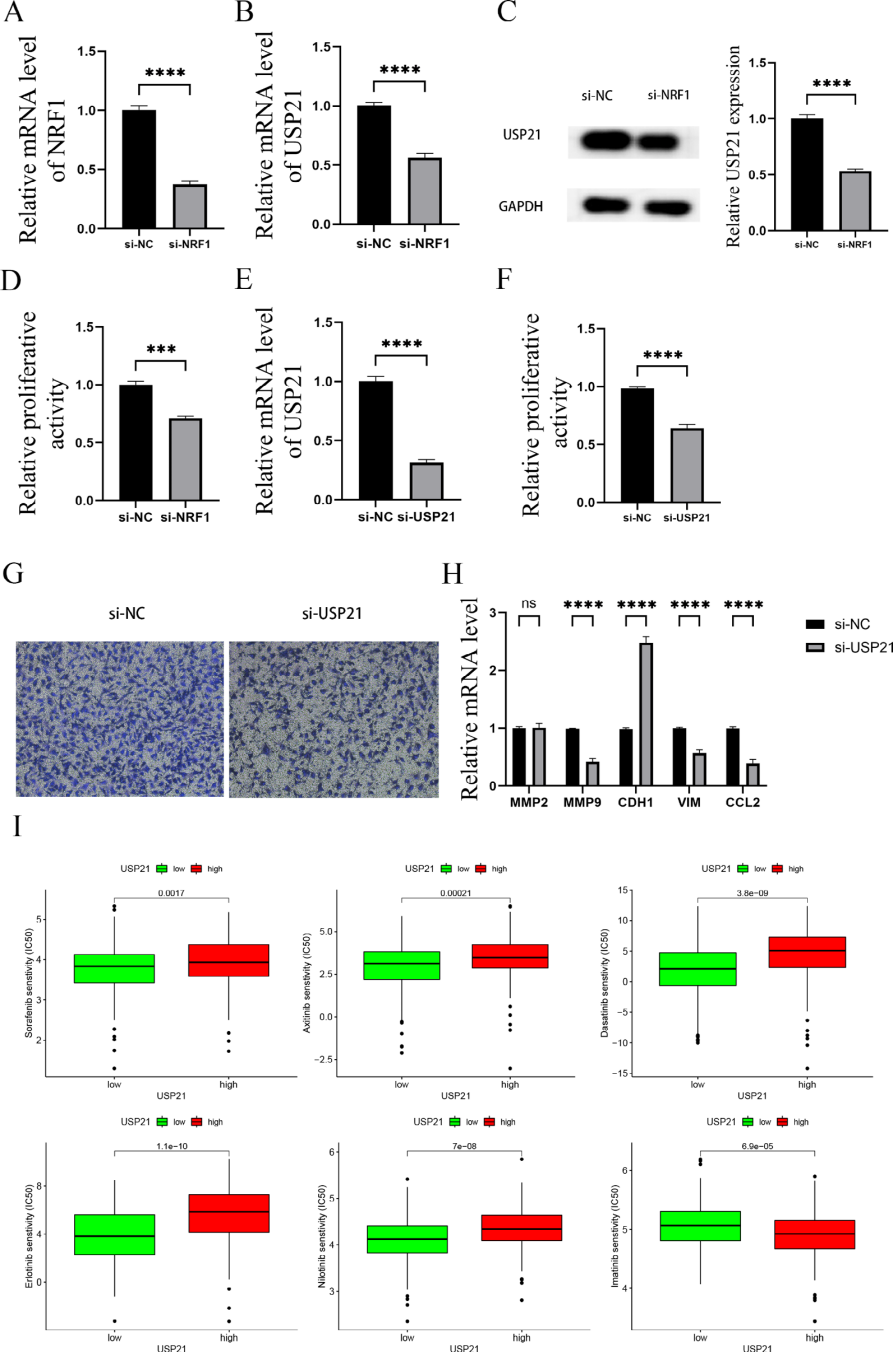
**NRF1 enhances Huh7 proliferation and migration through upregulating USP21 expression** (**A**) NRF1 mRNA expression in Huh7 cells after si-NRF1 transfection. (**B**) USP21 mRNA expression in Huh7 cells after si-NRF1 transfection. (**C**) USP21 protein level. (**D**) Effect of NRF1 knockdown on cell proliferation. (**E**) USP21 mRNA expression in Huh7 cells after si-USP21 transfection. (**F**) Effect of USP21 knockdown on cell proliferation. (**G**) Transwell assay, magnification: ×200. (**H**) Levels of MMP2, MMP9, CDH1, VIM and CCL2 in Huh7 cells after si-USP21 transfection. (**I**) Drug sensitivity test. ***p < 0.001, ****p < 0.0001, ns. no signification

To determine whether NRF1 regulates USP21 expression in HCC cells, NRF1 siRNA was transfected into Huh7 cells. The mRNA and protein levels of USP21 were reduced in cells with NRF1 knockdown (Fig. 9A-9C), indicating the regulation of NRF1 in mediating USP21 expression. In addition, knockdown of NRF1 inhibited cell proliferation in Huh7 cells (Fig. 9D). Interestingly, it was observed that USP21 knockdown also inhibited HCC proliferation (Fig. 9E and 9F). Knockdown of NRF1 or USP21 did not affect HL-7702 cells proliferation (Fig.S4G-S4J). Transwell migration assay demonstrated that USP21 knockdown inhibited HCC migration (Fig. 9G). Moreover, knockdown of USP21 reduced the levels of MMP9, VIM and CCL2 (Fig. 9H). Collectively, our data suggest that NRF1 may promote the proliferation and migration of HCC cell by upregulating USP21 expression. Due to the reason that USP21 expression may be correlated with the OS of HCC patients, then, drug sensitivity test between USP21 and six common molecular drugs (Sorafenib, Axitinib, Dasatinib, Erlotinib, Imatinib and Nilotinib) were performed (Fig. 9I). Our results suggest that patients with low USP21 expression are sensitive to Sorafenib, Axitinib, Dasatinib, Erlotinib and Nilotinib.

## Discussion

It is known that HCC imposes significant economic and medical burdens on societies worldwide. Effective biomarkers for monitoring HCC progression as well as treatment guidance are urgently needed [21–23]. In recent years, scholars have dedicated substantial efforts to exploring prognostic and therapeutic molecular markers for HCC. In addition, increasing studies have demonstrated the involvement of necroptosis in HCC progression [24–27]. In this study, we developed a 7-NRG prognostic signature and assigned Riskscores to HCC patients based on the prognostic signature computation formula via cox regression analysis. HCC patients were divided into the high- and low-risk subgroups according to the median of Riskscore. K-M survival analysis revealed that patients in high-risk subgroup had a shorter OS in comparison with those in low-risk subgroup (p < 0.05). It was observed that patients with advanced tumor grades and stages exhibited elevated Riskscores (p < 0.05). In addition, univariate and multivariate cox regression analysis identified Riskscore as an independent prognostic factor for HCC (p < 0.001). The differences in immune cell infiltration scores, PD-1 and PD-L1 expression levels, as well as tumor mutation burden between the high-risk and low-risk subgroups suggested that high-risk patients may be more suitable candidates for immunotherapy. Additionally, USP21, one of the 7-NRGs, was demonstrated to play a promotional role in HCC cell proliferation and migration. Drug sensitivity analysis revealed that patients with low USP21 expression were more sensitive to sorafenib compared to those with high USP21 expression. In summary, our signature may potentially improve HCC prognosis prediction and guidance in immunotherapy and drug treatment strategies tailored to different patients.

The immune system plays a pivotal role in eliminating tumor cells and distinct immune cells exert varying functions during this process. In this study, we found that patients with low Riskscores exhibited higher NK cell infiltration and better prognosis, which was consistent with the fact that higher levels of NK cell infiltration contribute to anti-tumor immunity [28, 29]. Conversely, patients with high-risk showed higher M2 macrophage infiltration, which has been reported to promote tumor growth, invasion, and metastasis by secreting various active substances [30]. In line with it, we found that patients with high M2 macrophage infiltration had a shorter OS. In addition, high-risk patients expressed higher levels of immune checkpoint PD-1 and CTLA4, suggesting that high-risk patients may benefit from immunotherapy targeting PD-1 or CTLA4 checkpoint inhibitors. However, it should be noted that our analysis results were derived from the TCGA cohort, which requires validation in more datasets and samples to ensure consistency.

This prognostic signature comprises 7 NRGs, namely HSP90AA1, HSP90AB1, PPIA, PGAM5, FAF1, USP21, and SQSTM1. Previous studies have demonstrated that HSP90A, PPIA, PGAM5, USP21, and SQSTM1 are involved in regulating HCC progression through distinct mechanisms [31–37], which supports the relevance of our prognostic signature to HCC progression. Given that USP21 exhibits the highest correlation with NRF1 (a TF), the preliminary verification of the regulatory relationship between NRF1 and USP21 was performed in HCC. We found that silencing NRF1 suppressed USP21 expression in HCC cells. Knockdown of NRF1 or USP21 inhibited HCC cell proliferation, whereas it had no impact on normal human liver cell (HL-7702) proliferation. Knockdown of USP21 was observed to suppress cell migration and reduce the mRNA expressions of MMP9, VIM, and CCL2 in Huh7 cells. Our findings suggest that NRF1 may influence HCC progression by regulating USP21. However, further validation are needed in more cell lines and animal experiments.

Previously, the prognostic value of necroptosis in cancers has been explored. For instance, Zhao et al. analyzed the prognostic values of necroptosis-associated lncRNA in stomach cancer [38]. Ren et al. constructed a 13-NRG signature for predicting HCC prognosis using univariate cox and lasso cox regression analyses [39]. However, these prognostic signatures were established only through analysis of public databases and lacked validation in clinical cohorts. Notably, our signature was not only validated in the TCGA and ICGC databases but also in the local cohort. Moreover, we validated the high expression of this 7-NRG signature in HCC tissues through immunohistochemical experiments.

## Conclusion

In conclusion, we developed a novel 7-NRG prognostic signature that could contribute to predict the HCC prognosis.

### Electronic supplementary material

Below is the link to the electronic supplementary material.

Supplementary Material 1

## Data Availability

The source data of this study were derived from the public repositories, as indicated in the section of “Materials and Methods” of the manuscript. And all data that support the findings of this study are available from the corresponding author upon reasonable request. The public data for the analysis in the present research could be obtained from the following websites: https://portal.gdc.cancer.gov , https://www.genome.jp/kegg , https://dcc.icgc.org.
