# Supplementary material for: Exploration and validation of a novel signature of seven necroptosis-related genes to improve the clinical outcome of hepatocellular carcinoma
Source: BMC Cancer. 2023 Oct 24;23:1029. doi: 10.1186/s12885-023-11521-x (PMC10594920; doi:10.1186/s12885-023-11521-x)

**Fig.S1** **Validation of 7-NRG signature in the local cohort**

**
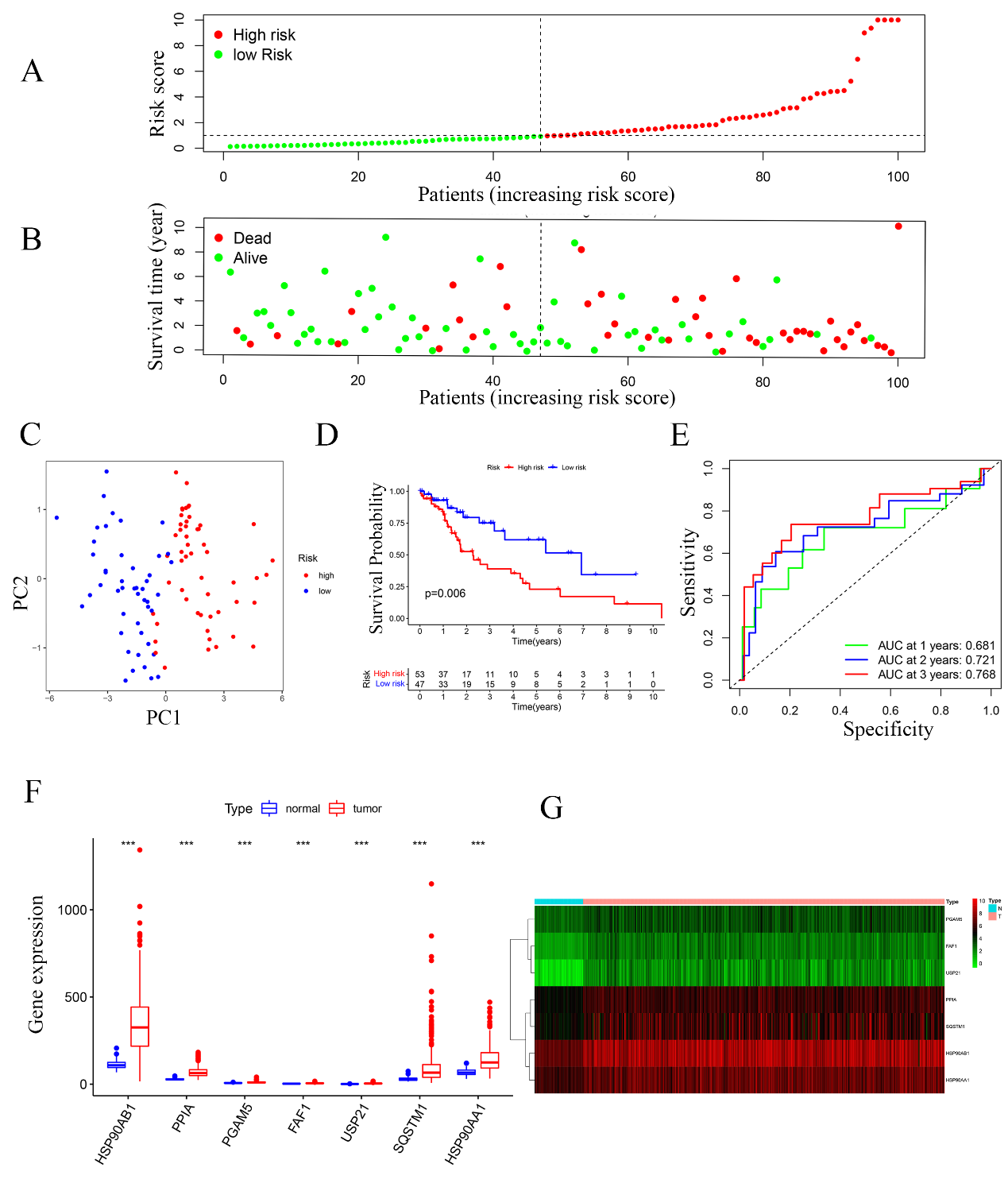
**

(A) Riskscores of HCC patients in the local cohort. (B) Survival status scatterplot in the local cohort. (C) PCA analysis in the local cohort. (D) Survival curves in the local cohort. (E) ROC curve analysis in the local cohort. (F) Differential expression analyses of seven NRGs in TCGA cohort. (G) Heatmap of differential expression analyses for seven NRGs in TCGA cohort.

**Fig.S2 Correlation analysis between Riskscore and clinical features**


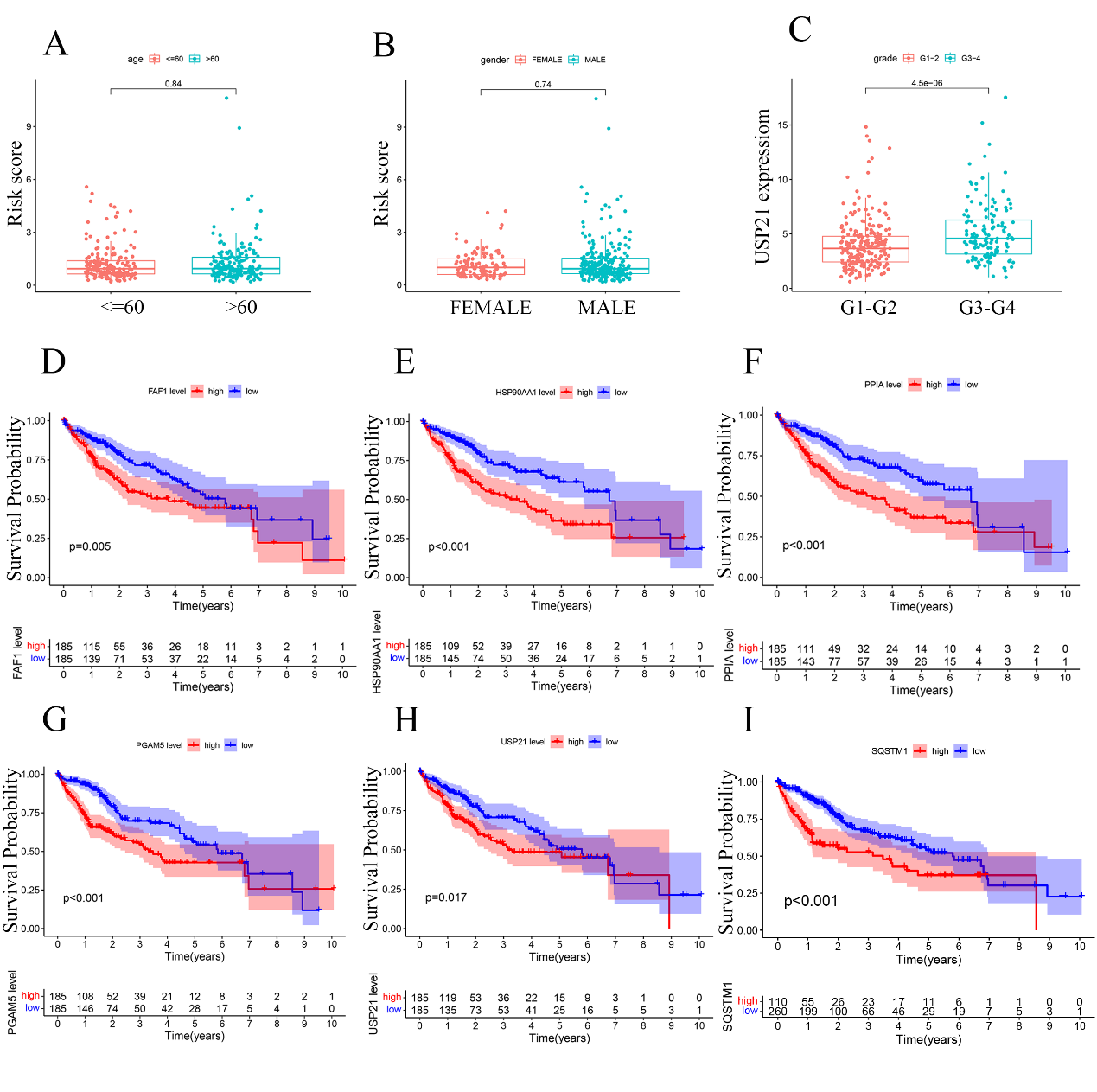


(A) The association between the Riskscore and age. (B) The association between the Riskscore and gender. (C) The association between USP21 expression and grade. (D-I) Survival curves in TCGA cohort.

**Fig.S3 Immunohistochemistry staining of PD-1 and PD-L1 from the local cohort.**

**
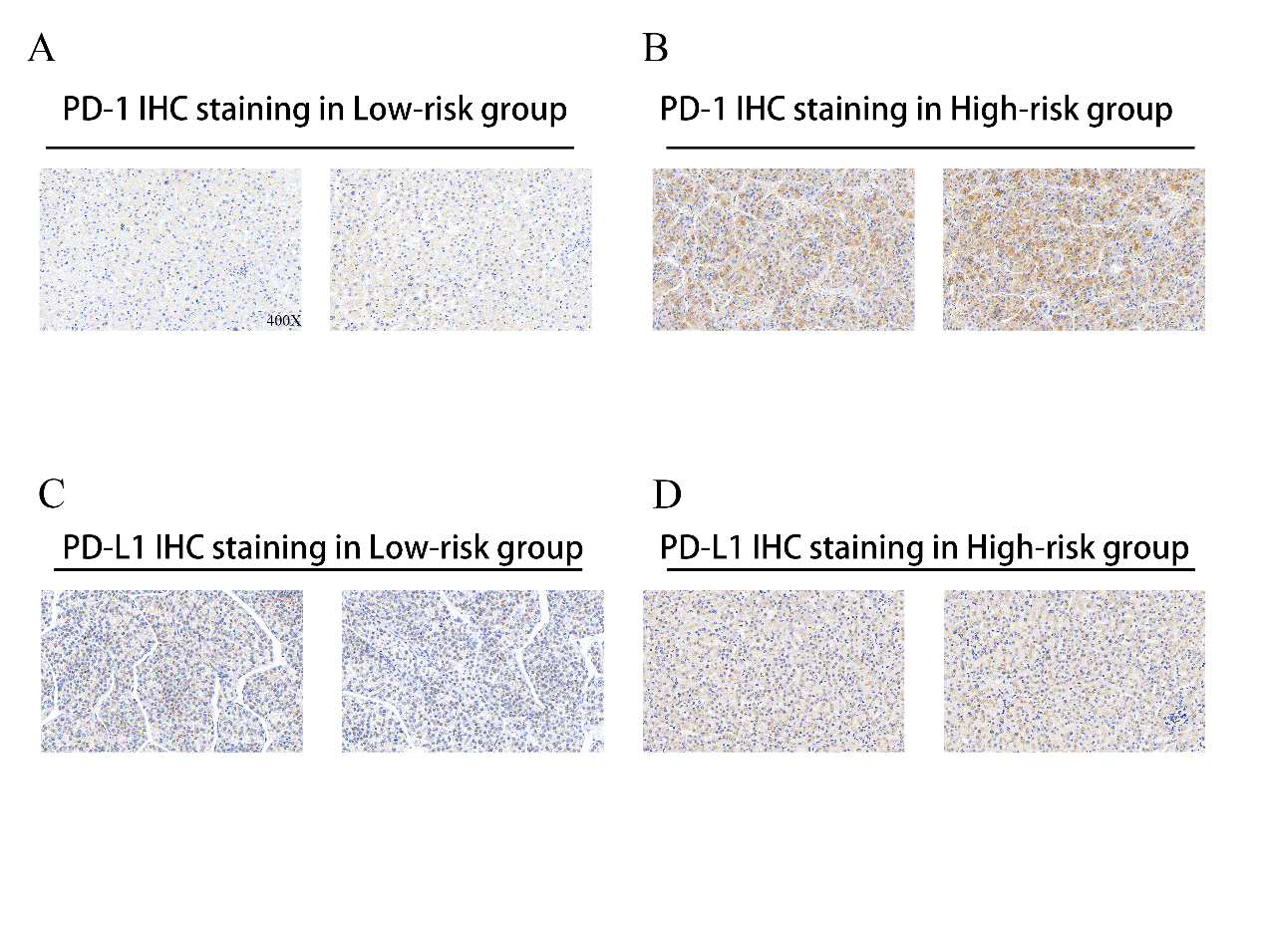
**

(A-B) Immunohistochemistry staining of PD-1. (C-D) Immunohistochemistry staining of PD-L1. Magnification: ×400.

**Fig.S4 Verification of upregulated NRF1 expression in ICGC and local cohort.**


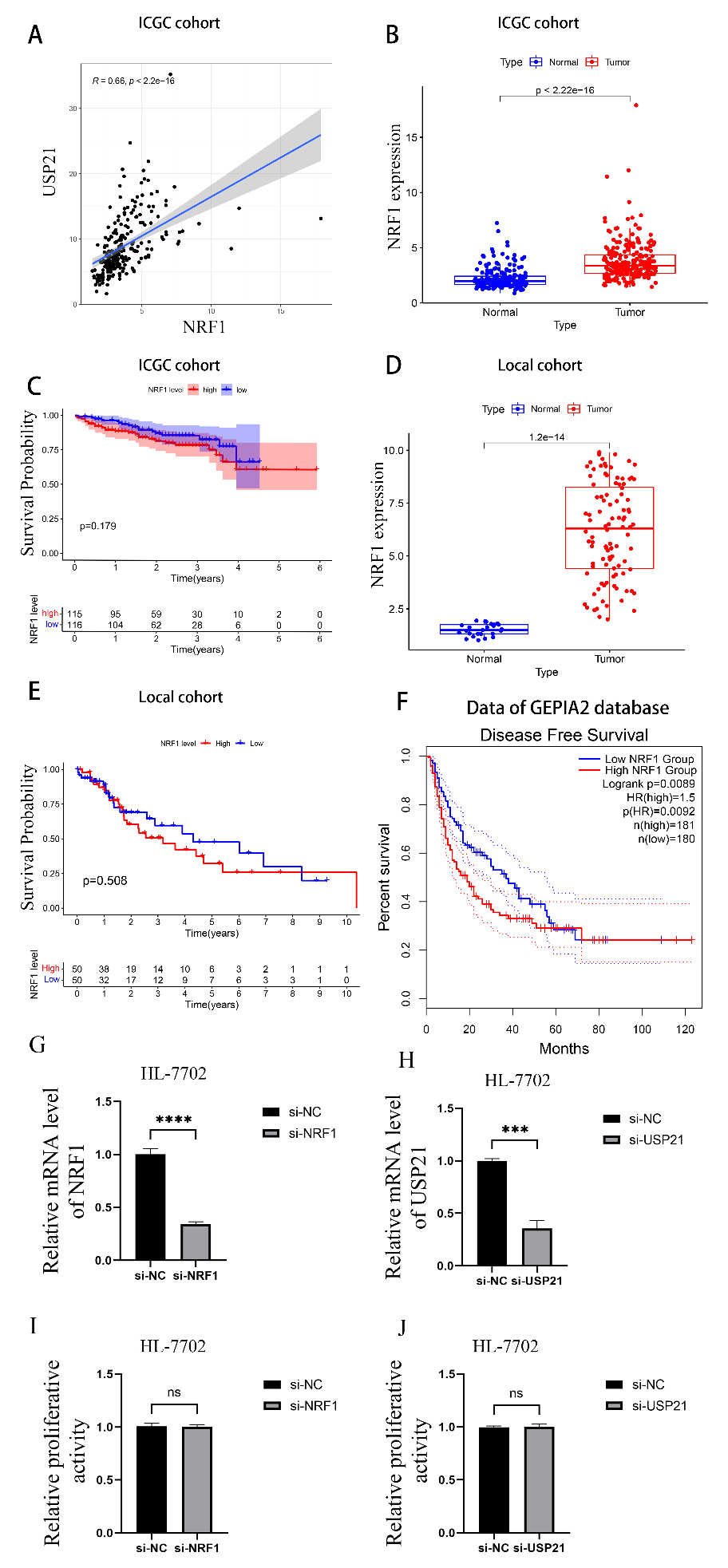


(A) NRF1 was positive correlated with USP21 in ICGC cohort. (B) NRF1 was upregulated in HCC tissues of ICGC cohort. (C) NRF1 survival curves in ICGC cohort. (D) NRF1 was upregulated in HCC tissues of local cohort. (E) NRF1 survival curves in local cohort. (F) NRF1 Disease free survival curve from GEPIA2 database. (G) NRF1 mRNA expression in HL-7702 cells after si-NRF1 transfection. (H) USP21 mRNA expression in HL-7702 cells after si-USP21 transfection. (I) Effect of NRF1 knockdown on HL-7702 cell proliferation. (J) Effect of USP21 knockdown on HL-7702 cell proliferation. ***p < 0.001, ****p < 0.0001, ns. no signification.

**Table S1:** Necroptosis-associated genes

| TNF | STAT1 | MAPK10 | H2AC21 |
| --- | --- | --- | --- |
| TNFRSF1A | STAT2 | MAPK9 | H2AZ2 |
| TRADD | STAT3 | FTH1 | H2AC7 |
| TRAF2 | STAT4 | FTL | H2AZ1 |
| TRAF5 | STAT5A | PLA2G4E | H2AC15 |
| RIPK1 | STAT5B | PLA2G4A | H2AC6 |
| BIRC2 | STAT6 | JMJD7-PLA2G4B | H2AC13 |
| BIRC3 | IRF9 | PLA2G4B | H2AC14 |
| XIAP | EIF2AK2 | PLA2G4C | H2AC16 |
| RBCK1 | TLR4 | PLA2G4D | H2AB2 |
| RNF31 | TICAM2 | PLA2G4F | PPIA |
| SHARPIN | TICAM1 | ALOX15 | BCL2 |
| SPATA2L | TLR3 | CAPN1 | IFNA1 |
| SPATA2 | ZBP1 | CAPN2 | IFNA2 |
| CYLD | USP21 | SMPD1 | IFNA4 |
| FADD | SQSTM1 | MLKL | IFNA5 |
| CASP8 | HSP90AA1 | PGAM5 | IFNA6 |
| CFLAR | HSP90AB1 | DNM1L | IFNA7 |
| RIPK3 | TNFAIP3 | NLRP3 | IFNA8 |
| CYBB | PARP1 | PYCARD | IFNA10 |
| CAMK2A | BID | CASP1 | IFNA13 |
| CAMK2D | BAX | IL1B | IFNA14 |
| CAMK2B | AIFM1 | CHMP2A | IFNA16 |
| CAMK2G | H2AX | CHMP2B | IFNA17 |
| SLC25A4 | H2AC20 | CHMP3 | IFNA21 |
| SLC25A5 | H2AC12 | RNF103-CHMP3 | IFNB1 |
| SLC25A6 | H2AC1 | CHMP4B | IFNG |
| SLC25A31 | H2AW | CHMP4A | IFNAR1 |
| PPID | H2AB3 | CHMP4C | IFNAR2 |
| VDAC1 | H2AC8 | CHMP6 | IFNGR1 |
| VDAC2 | H2AC4 | VPS4B | IFNGR2 |
| VDAC3 | MACROH2A2 | VPS4A | JAK1 |
| GLUD2 | MACROH2A1 | CHMP1B | JAK2 |
| GLUD1 | H2AC19 | CHMP1A | JAK3 |
| GLUL | H2AJ | CHMP5 | TYK2 |
| PYGL | H2AB1 | CHMP7 | TNFRSF10B |
| PYGM | H2AC17 | TRPM7 | FASLG |
| PYGB | H2AC18 | IL1A | FAS |
| MAPK8 | H2AC11 | IL33 | FAF1 |
| TNFRSF10A | HMGB1 | TNFSF10 |  |
|  | | | |

| Gene | Forward sequence | Reverse sequence |  |
| --- | --- | --- | --- |
| HSP90AA1 | 5'- AGGAGGTTGAGACGTTCGC -3' | 5'- AGAGTTCGATCTTGTTTGTTCGG -3' |  |
|  |  |  |  |
| PPIA | 5'- CCCACCGTGTTCTTCGACATT -3' | 5'- GGACCCGTATGCTTTAGGATGA -3' |  |
|  |  |  |  |
| SQSTM1 | 5'- GCACCCCAATGTGATCTGC -3' | 5'- CGCTACACAAGTCGTAGTCTGG -3' |  |
|  |  |  |  |
| HSP90AB1 | 5'- AGAAATTGCCCAACTCATGTCC -3' | 5'- ATCAACTCCCGAAGGAAAATCTC -3' |  |
|  |  |  |  |
| FAF1 | 5'- GAGATGATCCTGGCGGATTTTC -3' | 5'- AGGTCCTGGTATGGTCTCACC -3' |  |
|  |  |  |  |
| PGAM5 | 5'- TCGTCCATTCGTCTATGACGC -3' | 5'- GGCTTCCAATGAGACACGG -3' |  |
|  |  |  |  |
| USP21 | 5'-GAATCCTCGTGCTCCATCTGA -3' | 5'-CAGCTGGTATACAGGACTTCCG-3' |  |
|  |  |  |  |
| GAPDH | 5'- AAAGCCTGCCGGTGACTAAC -3' | 5'- GCCCAATACGACCAAATCAGA -3' |  |
| MMP2 | 5'- TGATGGCATCGCTCAGATCC -3' | 5'- GGCCTCGTATACCGCATCAA -3' |  |
| MMP9 | 5'- GGACAAGCTCTTCGGCTTCT -3' | 5'- TCGCTGGTACAGGTCGAGTA -3' |  |
| CDH1 | 5'- TACCCTGGTGGTTCAAGCTG -3' | 5'- CAAAATCCAAGCCCGTGGTG -3' |  |
| VIM | 5'- GGACCAGCTAACCAACGACA -3' | 5'- AAGGTCAAGACGTGCCAGAG -3' |  |
| CCL2 | 5'- CCCCAGTCACCTGCTGTTAT -3' | 5'- CTTCTTTGGGACACTTGCTGC -3' |  |

**Table S2** Primer sequences

**The uncropped gels image of WB image.**


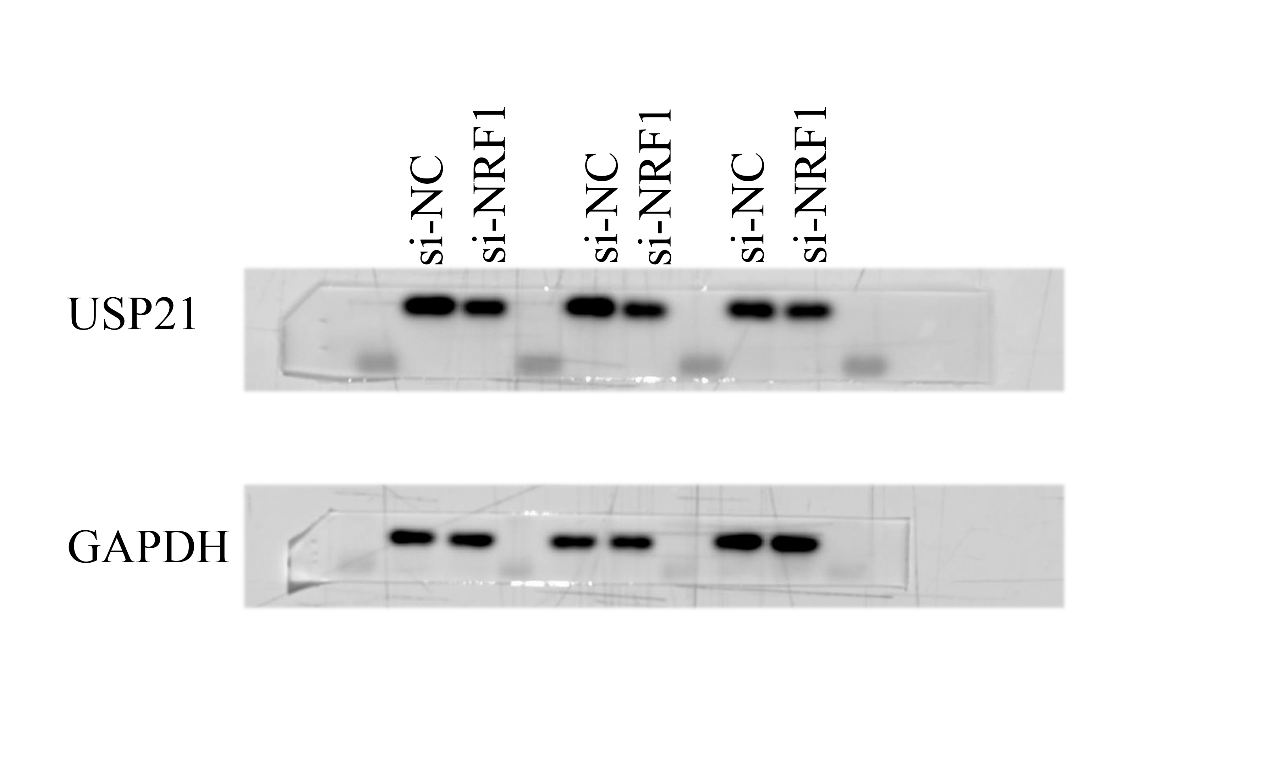

Supplement: Supplementary file 1 — Supplementary Material 1 [file 12885_2023_11521_MOESM1_ESM.docx]
